# The Community Prevalence of Vitamin C Deficiency and Inadequacy—How Does Australia Compare With Other Nations? A Scoping Review

**DOI:** 10.1002/hsr2.70943

**Published:** 2025-07-10

**Authors:** Danielle M. Carter, Hiep N. Le, Hai Phung

**Affiliations:** ^1^ General Practitioner Townsville Queensland Australia; ^2^ School of Medicine and Dentistry (Health Promotion) Griffith University Gold Coast Queensland Australia; ^3^ School of Medicine and Dentistry Griffith University Gold Coast Queensland Australia

**Keywords:** community prevalence, nutritional inadequacy, scurvy, serum ascorbate, vitamin C deficiency

## Abstract

**Background and Aims:**

Vitamin C is a vital nutrient for health and wellbeing and is essential for the prevention of and recovery from many conditions and diseases. This review aimed to map existing literature, identify knowledge gaps, and provide an overview of the community prevalence of vitamin C deficiency and inadequacy globally, based on serum levels. The Australian context is discussed in detail.

**Methods:**

A systematic search using Scopus, PubMed, and CINAHL was conducted for articles reporting on prevalence rates of vitamin C deficiency and/or inadequacy, with serum vitamin C levels, published from 2003 to 2023. Studies including hospitalised patients were excluded.

**Results:**

From 4842 unique studies identified, 107 met criteria for inclusion in the final review. Only 31 countries have conducted studies on community serum vitamin C levels in the last 20 years, with nationally representative studies lacking in all age groups, particularly in preschoolers, children, and adolescents (except for the US and Mexico). The prevalence of deficiency in high‐income countries is likely between 0% and 15%, with current reference ranges underestimating overall inadequacy for optimal health, by approximately 33.6%.

**Conclusions:**

Australia and other nations around the world may have high levels of undetected vitamin C inadequacy which requires urgent investigation. Outdated reference ranges used for measuring serum vitamin C levels in Australia likely underestimate the prevalence of inadequacy. Serum vitamin C levels should be added as a biomarker to the next Australian Health Survey to assess community prevalence of deficiency and inadequacy. Screening guidelines for vitamin C deficiency and inadequacy should be established for use by Australian General Practitioners. The Australian Recommended Dietary Intake for vitamin C should be increased to align with levels that promote optimal health outcomes, rather than merely prevention of deficiency.

## 1 Introduction

Vitamin C (VC) is a fundamental nutrient for health and wellbeing [1]. Humans rely solely on acquiring VC by dietary means, through the consumption of fruit and vegetables [1]. VC a vital component of multiple pathways for hormone production, collagen synthesis, immune response, and gene regulation [2]. In addition, VC has powerful antioxidant effects for controlling inflammation and, therefore, plays a role in chronic disease and cancer prevention [3, 4, 5, 6]. Inadequate intake and higher requirements due to body composition, illness, chronic disease states, behavioural and environmental factors results in deficiency [2]. VC status is a spectrum that ranges from deficiency (< 11.4 µmol/L) through to saturation (≥ 70 µmol/L), with adequate being defined as greater than or equal to 50 µmol/L in more recent literature [7]. In complete absence of dietary VC, a fully replete body can become severely deficient within 2 months, with a risk of scurvy [1].

VC levels are inversely correlated with blood pressure readings [8], cataracts [9], blood glucose levels [2], body‐mass‐index and metabolic syndrome [10, 11]. Coronary artery disease is 2.3 times more likely in people with VC deficiency than those with levels above 70 µmol/L [12]. Bone mineral density scores are significantly higher [13] and dental problems markedly lower [14], amongst those with adequate serum VC. Insufficient VC levels are also associated with depression [15], poor cognitive function [16, 17] and Alzheimer's disease [18], signifying the importance of this nutrient for holistic health. Risk factors for VC deficiency include male biological sex, lower socioeconomic status, homelessness, lower educational attainment, smoking, obesity, hospitalization, institutionalization, poor dietary intake, environmental pollution and displacement [5, 7, 11, 19].

VC deficiency is well documented in hospitalised patients, particularly in those with critical illness or undergoing haemodialysis [20]. United States (US) studies report population prevalence rates of inadequate VC levels of approximately 40% [7, 21]. A recent Australian study of hospitalised patients found that around 55% had serum levels below 40 µmol/L [22]. With only 6.8% of Australians consuming their 5 servings of vegetables and only half eating 2 servings of fruit daily, there is genuine potential for deficiency [23].

In Australia, to our knowledge, there have been limited studies on community VC levels performed in the past 20 years. Aboriginal and Torres Strait Islander peoples have significantly higher morbidity and mortality rates compared with non‐Indigenous Australians [24] and, based on known risk factors for VC deficiency, may be at increased risk of a myriad of conditions, that could be reduced through identifying low VC levels at routine health assessments. Developing an understanding of how Australia compares with other nations, and identifying gaps in research, can prompt investigation and assist in the development of appropriate screening pathways for detecting VC deficiency and inadequacy in General Practice settings, which to our awareness, do not currently exist. This may further aid in primary and secondary prevention of some chronic diseases that inflict significant health burdens on individuals and healthcare systems alike and provide opportunities for targeted nutritional advice with biochemical confirmation of a need for action, through changes in dietary consumption habits or supplementation when indicated.

## 2 Objectives

The aim of this review is to provide a contemporaneous overview of the community prevalence of VC deficiency and inadequacy globally based on serum VC levels, through mapping existing literature and identifying knowledge gaps. The prevalence of VC inadequacy is highlighted between countries, with specific reference to the Australian context to examine future research needs.

## 3 Methods

The current scoping review was designed according to the Joanna Briggs Institute protocol [25] in conjunction with the PRISMA‐ScR checklist [26] and registered on Figshare (https://doi.org/10.6084/m9.figshare.24610503.v2).

A database search was performed in November 2023 using Scopus, PubMed and CINAHL for human studies published between 1 January 2003 and 21 November 2023, in English. A grey literature search using Google, for articles by government and non‐government organisations, found no additional resources.

Search terms included: (prevalence OR incidence OR status OR population* OR frequency OR seroprevalence) AND ((scurvy OR “hypovitaminosis C”) OR ((“ascorbic acid” OR ascorbate OR “vitamin C” OR “vitamins C”) AND (deficien* OR inadequa* OR insufficien* OR deplet*))).

Search results were compiled in EndNote21 [27] and uploaded into Covidence [28], where duplicates were removed. Abstract and title screening were performed by 2 reviewers (DMC and NHL). Full text screening was performed by 1 reviewer (DMC) and checked by a second (NHL).

Eligible articles from online sources reported both the prevalence of VC deficiency and serum VC levels in study participants. No limits were applied to age. Case reports, case series, editorials, letters, textbooks, incomplete or draft reports were excluded. Interventional studies were only included if baseline VC levels were reported. Studies were excluded if participants were hospitalised or inpatients at time of blood collection, as the focus of this review is on VC inadequacy in community settings. Due to the quantity of studies identified, additional exclusion criteria were then applied to exclude any studies which included subjects who currently had cancer, haemodialysis, parenteral nutrition or were pre‐ or post‐operative surgical patients as these populations have been thoroughly assessed in other literature. Studies based on dietary assessment, mean or median VC levels in isolation, without prevalence rates, were not included.

Data extracted from articles for analysis are as follows: Authors, year of publication, study type, number of participants, age range, setting, mean serum VC, and percentage per category of VC status, if available. The settings were defined as:

### 3.1 Outpatient

Patient attending hospital clinic for specialist care whilst residing in the community, including antenatal and obstetric clinics conducted in a hospital.

### 3.2 Health Care Service

Participant attending primary health, preventative, antenatal or dental care services whilst residing in the community, not requiring access to specialist care, even if care is delivered on a hospital campus.

### 3.3 School

Study was performed at or recruitment of students attending a primary or secondary school for children and adolescents.

### 3.4 Community Level

A community‐based study assessing populations within a given region or regions, narrower coverage, and smaller numbers than national study.

### 3.5 National

Multiple communities or large numbers of individuals (> 1000) sampled across a nation to provide representative data that can be applied to the entire population.

Serum VC reference ranges and units of measure differed widely throughout the literature. Units of measures were converted to equivalent µmol/L values using an online calculator [29].

This study used 5 reference range categories of VC status based on those described by Crook et al. [7], although also incorporating older cut‐off points. These were: Deficiency (< 11 or 11.4 µmol/L), Hypovitaminosis (11–23 or 28 µmol/L), Inadequate (23 or 28–49.9 µmol/L), Adequate (50–69.9 µmol/L) and Saturating (≥ 70 µmol/L). Due to variability in defined cut‐offs throughout the literature, studies not using Crook's criteria were categorised in a “best‐fit” manner, with reference ranges included, if available.

Studies were mapped based on country of research, to identify which global populations and subgroups have been assessed for VC deficiency and inadequacy (Table 1).

**Table 1:** Age groups studied per country.

High income countries^a^	0–5 yrs	6–12 yrs	13–18 yrs	19–60 yrs	60+ yrs
Australia [16, 30, 31, 32]					
New Zealand [17, 33]					
US [3, 4, 7, 11, 12, 13, 21, 34, 35, 36, 37, 38, 39, 40, 41, 42, 43, 44, 45, 46]					
Canada [47, 48, 49]					
Germany [50, 51, 52, 53]					
UK [54, 55, 56, 57, 58, 59, 60, 61]					
Austria [62, 63]					
Poland [64]					
Denmark [65]					
Slovakia [66]					
The Netherlands [67, 68]					
France [69]					
Switzerland [70]					
Japan [71]					
Upper middle income^a^					
Mexico [72, 73, 74, 75, 76]					
Cuba [18]					
Brazil [77, 78, 79, 80, 81, 82]					
Ecuador [83, 84]					
Venezuela [85]					
Thailand [86, 87, 88, 89]					
Taiwan [90, 91]					
China [92, 93, 94]					
Malaysia [95]					
Lower middle income^a^					
Papua New Guinea [96]					
Nigeria [97, 98, 99, 100, 101, 102, 103]					
India [9, 77, 104, 105, 106, 107, 108, 109, 110, 111]					
Bangladesh [112, 113, 114, 115, 116]					
Sri Lanka [117]					
Indonesia [14, 118]					
Philippines [19]					
Low income^a^					
Uganda [119]					
Gambia [120]					
Central African Republic [121]					

	Nationwide population study
	≥ 2 large (≥ 400^b^) community/outpatient/schools/health care services studied
	1 large (≥ 400^b^) community/outpatient/schools/health care services studied
	≥ 2 small (< 400^b^) community/outpatient/schools/health care services studied
	1 study not fitting other criteria
	Gaps in reported prevalence research

^a^ World Bank, 2018 [122].

b Participants with serum vitamin C levels.

The prevalence rates of deficiency, hypovitaminosis C and inadequacy were summarised per country and provided as a range, based on the lowest and highest reported values in relevant countries. Excluded were studies or groups of participants with known chronic diseases, small community studies with less than 80 subjects or with significantly high prevalence rates and studies with vastly different reference ranges. Whilst this is not highly reliable or accurate, it offers an approximate estimate of community prevalence rates to identify the need for further investigation (Table 2). For single‐study prevalence rate, the value has been used as a minimum prevalence estimate. When studies used a slightly different reference range, these are noted in Table 2 for transparency.

**Table 2:** Summary of serum vitamin C status per country.^a^

Countries per income group [123]	Summary of serum vitamin C status per country^a^		Estimated % inadequate (> 23 or 28–< 50 µmol/L)	References
	Estimated % deficiency (< 11 or 11.4 µmol/L)	Estimated % hypovitaminosis C (11 or 11.4–23 or 28 µmol/L)		
High income countries				
Australia	3.75	12.5	Nil data	[30]
New Zealand	2.4	10	51.6	[17]
United States of America	4.9–15.1	9.4–22.2	23.8–28.2	[3, 7, 12, 21, 35, 36, 37, 40, 42, 43, 44]
Canada	< 3–14	26–33	Nil data	[47, 48, 49]
Germany	0–3.3	14.1	11.4–20	[50, 51, 52]
United Kingdom	< 1–33	10.7–21	Nil data	[56, 57, 58]
Poland	0.8	6.7	67.4 (29–55 µmol/L)	[64]
Slovakia		11.23 (< 23 µmol/L)	42.25	[66]
Upper middle income				
Mexico	2.9–40	28.4–38.1	Nil data	[72, 75, 76]
Brazil	7.5–31	Nil data	Nil data	[77]
Ecuador	32.6–82	Nil data	Nil data	[83, 84]
Venezuela			34.4–62.3 (< 51.1 µmol/L)	
Thailand	8–44	16–38 (11–22 µmol/L)	Nil data	[86, 88]
Taiwan	1	9.8 (11.92–34.07 µmol/L)	28.1 (34.07–50 µmol/L)	[91]
China		21–65.5 (< 22.71 or 28 µmol/L)	Nil data	[92, 93, 94]
Lower middle income				
Nigeria		40–79.5 (< 28.39 and 31.23 µmol/L)	Nil data	[99, 100, 101]
India	6.3–73.9	15.3–66.1	Nil data	[9, 77, 105, 106, 107, 109, 110]
Bangladesh	2	9.6	Nil data	[113]
Indonesia	11.2–14.7	13.8–33.7 (11.36–22.14 µmol/L)	Nil data	[14, 118]
Low income				
Uganda	25–70.2	28.9–63.5	Nil data	[119]
Gambia	9	Nil data	Nil data	[120]
Central African Republic	15–42.2	10.8–33.4	Nil Data	[121]

^a^ Excludes participants with known chronic disease reported in study, e.g., inflammatory bowel disease, studies with less than 80 participants and those using vastly different reference ranges for deficiency, hypovitaminosis or inadequacy.

## 4 Results

### 4.1 Study Characteristics

**Figure 1:**
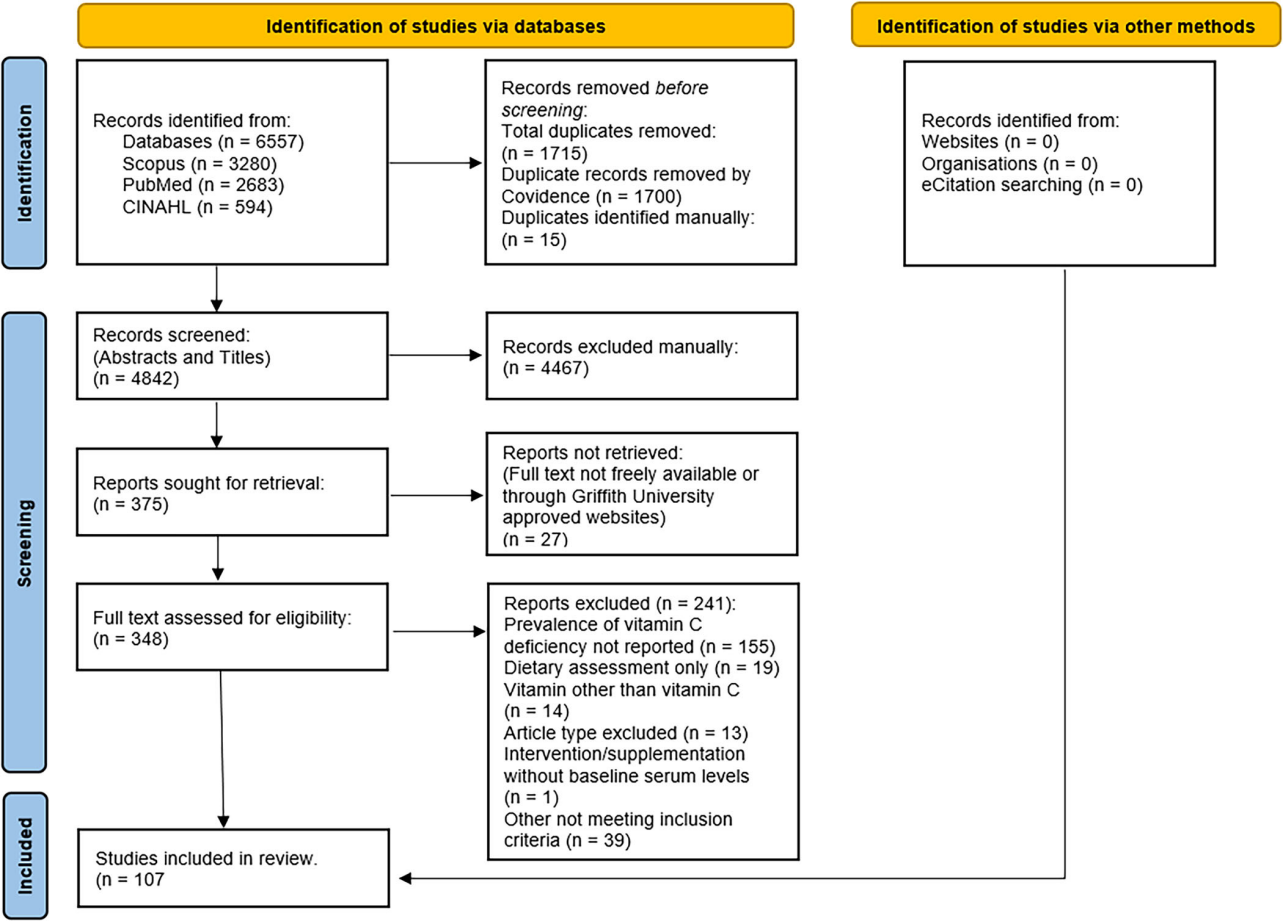
PRISMA flow diagram [124].

A total of 107 studies, from 31 countries were included in the final analysis, further described in Figure 1. Cross‐sectional design accounted for 70% of studies, 11% were randomised control trials and 7% were case‐control and prospective cohort studies, respectively. The remaining 6.5% included four retrospective cohort studies, two longitudinal studies and one report. Complete tabulated extraction data available in Appendix A.

Eighteen percent of studies (20/107) were conducted in the US using data from the annually conducted and population‐representative National Health and Nutrition Examination Surveys (NHANES) [12]. Ten studies were undertaken in India, eight in the United Kingdom (UK), seven in Nigeria, six in Brazil and four in Australia.

Population size varied widely from 8 to 52,150 subjects [34, 35]. One study did not report the number of participants [104]. Almost half (42%) of the studies had 200 or less participants. The most common settings for studies were at community level, national and outpatient clinics, occurring in 32, 27, and 21 percent of studies, respectively. Case‐control studies (*n* = 6) were not included in this calculation due to multiple settings being used. Schools (12.9%) and Health Care Services (7.9%) were less frequently studied.

Female subjects were studied more often than male subjects. Participants of two studies were solely male, whilst 21 studies were purely on female subjects, 6 of which included pregnant females.

National prevalence studies in adults have been conducted in the US [3, 7, 11, 12, 21, 35, 36, 37, 38, 39, 40, 41, 42, 43, 44], Canada [47], Mexico [72, 73, 74], and the UK [54, 55, 56, 57, 58], with people aged 60 years and older being assessed in the US [3, 7, 11, 12, 21, 35, 36, 37, 38, 39, 40, 41, 42, 43], Canada [47], the UK [54, 55, 56, 57, 58], India [9, 77] and Thailand [86]. The serum prevalence of VC deficiency and inadequacy in children and adolescents at a population‐wide level has been undertaken in the US [3, 39, 40, 43] and Mexico [72, 73, 74]. Two large community studies on girls aged 10–18 years [105, 106], and 3 other studies involving male and female children, and adolescents have been undertaken in India [104, 107, 108] (Table 1). One large community study including adolescents 14–18 years [125] and a small study on people aged 10–20 years with obesity [78] were performed in Brazil.

Australia had 4 studies reporting serum VC levels. Two studies used the same dataset with 80 subjects [16, 30], while the remaining studies had small sample sizes of 11 and 20 adults [31, 121]. No large or population prevalence studies conducted in Australia in the past 20 years.

### 4.2 Gaps in Research

The serum prevalence of VC deficiency and inadequacy in the under 5 years age group is unknown globally, with the exception of the US and Mexico. Children (6–12 years) are also remarkably understudied, particularly at the national level. In the studies identified, large‐scale community and national serum prevalence studies were lacking in the majority of countries, in all age groups (Table 1).

### 4.3 VC Status

Fifty‐eight studies reported on the prevalence of severe VC deficiency (< 11.4 µmol/L). In New Zealand, Pearson et al. [17] and Wilson et al. [33] found very low prevalence rates of deficiency in adults of 2.4 and 0%–4%, respectively. Other studies conducted in Germany [50, 51], Poland [64], The Netherlands [67], Cuba [18], the UK [56, 57, 59], Taiwan [90, 91], and Nigeria [97] found similar rates. High income countries had the lowest rates of deficiency (Table 2). The highest rates of deficiency were seen in pregnant women in Uganda and the Central African Republic [119, 121].

Rates of deficiency varying from 4% to 15% were reported in Australia [30, 32], the US [7, 21, 35, 36, 37, 39, 40], Canada [47, 48, 49] (Table 2). Markedly high deficiency rates of more than 30% were found in certain populations, including the elderly and low income households in the UK [58, 108], prisoners in Papua New Guinea [96], people living with HIV in the US [8], and the elderly, and people living in slums in both India and Ecuador [9, 83, 110]. Higher rates were often seen in obesity and chronic disease in children, adolescents and adults [4, 75, 79, 87]. In the majority of studies, males experienced deficiency to a greater degree than females.

Inadequate serum VC levels are far more common throughout the world than deficiency, however, comparable data is not available for many countries due to cut‐offs of 23 or 28 µmol/L being used. Estimated rates of inadequacies reported in literature from 2003 to 2023 are presented in the Table 2. Large population studies in the US found around 42% of the population have serum levels < 50 µmol/L [12, 21]. In the UK, 35.6% males and 17% females, aged 40–79 years have inadequate VC levels [56]. In older people living in India, 100% of the population had levels below 52.5 µmol/L [9]. Australian studies used different cut‐offs, however, 30% of patients attending a dental clinic were found to have levels under 40 µmol/L [32], whilst another study undertaken in a privately billing integrative medicine practice reported 16.5% of participants had levels less than 28 µmol/L [16, 126]. Subjects with obesity, metabolic syndrome and diabetes frequently had prevalence rates of more than 60% for inadequate VC levels [31, 33, 78, 127].

The final finding was that using cut‐offs of 23 or 28 µmol/L to classify inadequacy underestimates a large proportion of the population in these studies who have inadequate VC levels (< 50 µmol/L) for optimal health outcomes, by approximately 33.6% (range 25%–51.6%) (Table 3). This review selected studies which used 5 categories of VC status [7], with unique data sets, with 50 µmol/L as the accepted “normal” or adequate VC reference point to determine what percentage of people in these studies would be overlooked if a lower cut‐off (23 µmol/L) were used. Findings would be similar but slightly lower if 28 µmol/L were to be used.

**Table 3:** Percentage of population sub‐categories of vitamin C status.

Country	< 11 µmol/L	11–23 µmol/L	23.1–50 µmol/L	50.1–69.9 µmol/L	≥ 70 µmol/L
New Zealand [17]	2.4	10	51.6	29	7
United States [12]	6.1	9.5	26.2	32.4	25.8
United States [21]	8.4	9.4	28.2	27.8	26.1
United States [44]	14.8		25	32.6	27.6
Slovakia [66]	11.23		42.25	46.52	
Taiwan [91]	1	9.8	28.1	56	5.1
Average			33.6		

## 5 Discussion

This review discovered multiple findings relevant to public health nutrition. Only 31 countries have conducted studies on community serum VC levels in the last 20 years, with national representative studies lacking in all age groups, particularly in preschoolers, children and adolescents (apart from the US and Mexico). According to data from the Global Burden of Disease Study 2017, “consumption of nearly all healthy foods and nutrients was suboptimal” [128] ^(p1961)^, with all countries falling grossly short of recommended fruit intake and all regions, except central Asia, consuming insufficient vegetables [128]. When monitoring consumption habits, dietary assessments are prone to inaccuracy due to recall bias, social approval bias and limitations in those who frequently consume food outside of the home [129, 130].

Median serum VC levels used at a population level can be falsely reassuring and ignore groups suffering from serious deficiency [2]. In adults, serum VC is a preferred biomarker for fruit and vegetable intake due to it being an objective measure; although evidence is conflicting for its use in children and adolescents [131]. Whilst serum VC levels are vulnerable to pre‐laboratory degradation, and oxidative stress, when done as fasting measures using high‐performance liquid chromatography (HPLC) technique they are a reliable way of assessing deficiency and inadequacy [7, 130]. In some settings, particularly where access to laboratory processing is delayed, the accuracy of serum VC levels may be reduced, which needs to be considered in the interpretation of results [2].

Given that intakes of VC rich foods are insufficient globally, and the VC status of the world population in community settings is largely unknown, there is an urgent need for national prevalence studies, not just to assess deficiency but also to identify populations at risk of a multitude of chronic health problems, inflammation and immune dysfunction due to inadequate serum levels [3, 5, 10, 132]. The three leading causes of death due to non‐communicable diseases worldwide include cardiovascular disease, cancer and chronic respiratory diseases [133], components of which have shown correlation with VC levels [2, 12, 42, 50, 87, 94], reinforcing the need for further investigation into VC inadequacy as a preventable risk factor. In Table 3, the present study compared the cut‐offs of 23 µmol/L (defining hypovitaminosis C) and 50 µmol/L (defining inadequacy for ideal health outcomes). This comparison revealed a 33.6% higher prevalence of inadequate levels when using 50 µmol/L as the cut‐off. Therefore, using 23 µmol/L as the cut‐off underestimates the number of people at higher risk of poor health outcomes. It is recommended that future prevalence studies adopt additional cut‐offs, including 50 µmol/L for inadequacy, to better categorise VC status.

The prevalence of both deficiency and inadequacy were generally higher in outpatient settings compared with national populations and other community settings, but lower than rates observed in hospitalised patients in high‐income countries, where deficiency (< 11.4 µmol/L) was found in around 27% of patients [101]. This is likely due to the existence of chronic illnesses, a known risk factor for low VC levels, although not reaching the degree of deficiency seen in acute severe illness in hospital inpatients. Earlier studies on adults in France and Singapore in the 1990s, identified deficiency rates of 1%–9% and 12%, respectively [2]. Finland reported a total of 6.6% (< 28 µmol/L) during the same period, while studies in Spain and Austria reported median VC levels of 45 and 58 µmol/L [2], respectively. These findings were consistent with the current results, however, also may suggest that there has been no improvement in VC status between the 1990s and 2023 in high‐income countries.

Pregnant females were excluded from many national studies. In African studies, pregnant females had significantly low VC levels as a group and compared with non‐pregnant females [119, 121, 123]. Pregnancy demands a higher intake of VC to satisfy maternal and foetal requirements [132]. One study found a correlation between maternal deficiency and infant deficiency during the first 6 months post‐partum [121]. Further studies on VC levels during pregnancy and infancy may add valuable insights into potential factors contributing to long‐term health outcomes.

Most studies were cross‐sectional in design, which is the ideal type for assessing population prevalence of a condition [134]. Other study types were included in this review to broaden results, such as using baseline serum VC levels in randomised controls trials or in voluntary community participants in case‐control studies. This has assisted in identifying gaps in current knowledge of community VC status where large cross‐sectional studies have not been performed.

### 5.1 Australian Context

There are limited Australian studies on VC levels. Four community studies were identified, each with small sample sizes between 11 and 80 adults [16, 30, 31, 32]. Estimated prevalence ranges are similar to those found in adults in the US, where around 40% of the population have inadequate VC levels [12]. In recent years, an Australian study on hospitalized patients has discovered rates of 24.5% deficiency and 29.9% hypovitaminosis, totalling 54.4% having serum levels less than 40 µmol/L [22]. A study of medical inpatients found 76.5% had hypovitaminosis C (< 28 µmol/L), with 41.6% being deficient [134]. One study on adult surgical outpatients was not included in the current study due to some participants having cancer (exclusion criteria), however, 43% of patients had levels below 28.4 µmol/L, of whom 21.4% were deficient (< 11.4 µmol/L) [135]. Levels below 28 µmol/L were present in 61.9% of people 75 years and older in another hospital study [136].

Australian Recommended Dietary Intake (RDI) for VC remains at 45 mg/day for men and women aged 19 and older, with the Estimated Average Requirement (EAR) of 30 mg/day [137]. According to the Australian Bureau of Statistics [138], “less than 5% of the population had inadequate intake of (VC) based on EAR.” This is in agreeance with an Australian Institute of Health and Welfare report, which states the nutrient intake of Australians is not “adversely affected” [139, p. ix] by consuming less than recommended portions of fruits and vegetables. These statements are based on dietary assessments alone using an EAR and are in stark contrast to the findings of Australian and global studies on VC levels and prevalence rates of deficiency. Only 68% of Australian children and adolescents consume RDI of fruits, whilst 95% of Australian children and adolescents do not consume enough vegetables [140]. Nationally, proportions meeting RDI for fruit and vegetable consumption are 54% and 6.8%, respectively [138]. In light of higher cut‐off points discussed for adequate VC levels and other authorities recommending increased dietary intakes of 110 mg [2, 132], Australia urgently needs to assess the serum VC status of its population and alter dietary guidelines as appropriate. The Australian Health Survey currently assesses some biomarkers (vitamin D, folate, vitamin B12, iron and iodine) to monitor nutritional intake and deficiencies [139]. To establish a true understanding of VC status, it is recommended that Australia include the use of fasting serum VC levels as a biomarker in the next Australian Health Survey.

### 5.2 Public Health Significance and Implications for Public Health Practice

Screening guidelines for detecting VC deficiency and inadequacy are required in Australia. Currently, serum VC is not referenced in the “Guidelines to Preventative Activities in general practice” [141] and is only mentioned in the “National Guide to a Preventive Health Assessment for Aboriginal and Torres Strait Islander People” [142] in relation to iron deficiency. The next Australian Health Survey should include fasting serum VC levels to assess the prevalence of deficiency and inadequacy in the Australian population. Currently, the Australian Health Survey includes biomarkers for iodine, folate, vitamin B12 and iron, but not specifically for VC [139]. Given, the importance of VC in preventing diseases and maintaining overall health, establishing screening guidelines for deficiency and inadequacy could allow early detection and treatment of deficiencies, ultimately improving public health outcomes. Healthcare professionals could use these guidelines to identify individuals at risk and provide targeted nutritional advice for “food as medicine” and supplementation when indicated. Moreover, public health policies could be informed by the prevalence data, potentially leading to initiatives aimed at improving the dietary habits of Australians. The results from the survey could also help to guide future research and health promotion activities related to nutrition in Australia and could be a valuable step towards improving the nutritional surveillance system in the country.

## 6 Limitations

An assessment of the quality of studies included was not within the scope of this review and a meta‐analysis was not performed. Therefore, the estimates provided are not intended to be an accurate measure but instead to represent possible prevalence rates and areas for future research. It is feasible that due to using a maximum of 3 databases and the specified inclusion criteria, that some prevalence studies performed over the last 2 decades may have been missed. Included articles only published in English may have limited results from countries where English is not widely used. It is also acknowledged that serum VC levels are vulnerable to oxidation [130] and may affect the accuracy of measurement in the included studies.

## 7 Conclusions

The community prevalence of serum VC deficiency and inadequacy is largely unknown due to a limited number of national and large community‐level studies being performed globally over the past 20 years. Studies in infants, children and adolescents are particularly scarce. A total of 50 µmol/L should be adopted as the lower limit of normal for serum VC levels. The prevalence of deficiency in high‐income countries is likely between 0% and 15%, with current reference ranges underestimating overall inadequacy for optimal health, by approximately 33.6%. Australia potentially has a high level of undetected VC inadequacy in the community which requires investigation.

## 8 Implications for Research

Further research is urgently needed in all nations across the life course (except for the US and Mexico) to determine VC status, using fasting serum VC levels, within community populations. Future studies should be encouraged to report on serum levels according to 5 categories of VC status described by Crook et al. [12], to gain a deeper understanding of the relationship between chronic disease risk and degrees of VC depletion.

## Author Contributions

Conceptualisation: Danielle M. Carter, Hiep N. Le, and Hai Phung. Methodology: Danielle M. Carter. Screening: Danielle M. Carter and Hiep N. Le. Formal analysis: Danielle M. Carter. Writing – original draft preparation: Danielle M. Carter. Writing – review and editing: Danielle M. Carter, Hiep N. Le, and Hai Phung. Supervision: Hiep N. Le and Hai Phung.

## Conflicts of Interest

The authors declare no conflicts of interest.

## Transparency Statement

The lead author Danielle M. Carter, Hai Phung affirms that this manuscript is an honest, accurate, and transparent account of the study being reported; that no important aspects of the study have been omitted; and that any discrepancies from the study as planned (and, if relevant, registered) have been explained.

## Data Availability

The data that support the findings of this study are available from the corresponding author upon reasonable request.

## Appendix A

See Table A1.

**Table A1:** Data extraction table.

Country	Authors	Year of publication	Study type	Population	Setting	Mean serum vitamin C (SD) (µmol/L)	% < 11 or 11.4 µmol/L	% 11 or 11.4–23 or 28 µmol/L	% 23 or 28–49.9 µmol/L	% 50–69.9 µmol/L	% ≥ 70 µmol/L	References
Oceania												
Australia	Christie‐David & Gunton	2017	Retrospective cohort study	Every patient attending urban diabetes clinic who had vitamin C levels tested. 11 adults, 42–68 years.	Diabetes clinic (outpatient)	Nil data [median 19]	64 [< 40]			36 [> 40]		[31]
Australia	Munday et al.	2020	Cross‐sectional Study	20 English‐speaking adults attending Westmead Centre of Oral Health Periodontic Clinic.	Dental clinic (health care service)	Nil data	10%	20 [11.4 < 40]		70 [≥ 40]		[32]
Australia	Travica et al.	2019	Cross‐sectional Study	80 adults. 24–95 years recruited from the National Institute of Integrative Medicine (NIIM).	General practice and health clinic (health care service)	[≥ 28] 53.09 (19.77) [< 28] 16.27 (6.45)	Total 16.25; M/F 26/11 [< 28]		Total 83.75; M/F 74/89 [≥ 28]			[16]
Australia	Travica et al.	2020	Pilot Cross‐Sectional study retrospective analysis.	80 adults. 24–95 years recruited from the National Institute of Integrative Medicine (NIIM).	General practice and health clinic (health care service)	M 44.46 (4.43) F 48.45 (3.13)	3.75	Total 12.5; M/F 26/11	M/F 74/89 [≥ 28]			[30]
Papua New Guinea	Gould et al.	2013	Cross‐sectional Study	148 male prisoners (representing 58.3% of total prisoners at facility), 13 male prison guards. ≥ 18 years.	Prison (community)	Nil data [median Prisoners 6.3, Guards 48.5]	Prisoners 64	Nil data	Nil data	Nil data	Nil data	[96]
New Zealand	Pearson et al.	2017	Prospective cohort study. Part of CHALICE study.	404 free‐living participants in Cantebury. 49–51 years. 369 has fasting vitamin C levels collected.	Population 50 years (community)	44.2	2.4	10	51.6	29	7	[17]
New Zealand	Wilson et al.	2017	Cross‐sectional study	89 participants. ≥ 18 years. Recruited from community‐based health services, pharmacies and media. Metformin only permitted.	Population (community)	Total 49 (17), Normal 57 (14), Prediabetes 48 (16), T2DM 41 (18)	Normal 0, Prediabetes 4, T2DM 3	Normal glucose 3, Prediabetes 0, T2DM 14	Normal 21, Prediabetes 58, T2DM 52	Normal 76, Prediabetes 36, T2DM 31 [> 50]		[33]
North America												
United States	Bird et al.	2017	Cross‐sectional study. Secondary analysis of NHANES survey (2003–2004, 2005–2006)	15,030 civilian, non‐institutionalized subjects. ≥ 9 years.	Population (national)	Nil data	2003–2004 = 7.5 2005–2006 = 4.9 2003–2006 = 6.2	Nil data	Nil data	Nil data	Nil data	[36]
United States	Blumberg et al.	2017	Cross‐sectional study. Secondary analysis of NHANES survey (2009–2010, 2011–2012)	10,698 civilian, non‐institutionalized subjects, ≥ 19 years. Pregnant and/or lactating females excluded.	Population (national)	Nil data	10.5 Population not taking supplements.	Nil data	Nil data	Nil data	Nil data	[37]
United States	Crook et al.	2021	Cross‐sectional study. Secondary analysis of NHANES survey (2003–2004, 2005–2006)	7607 participants. ≥ 20 years.	Population (national)	54.63 (28.62)	6.1	9.5	26.2	32.4	25.8	[7]
United States	Crook et al.	2022	Cross‐sectional study. Secondary analysis of NHANES survey (2003–2004, 2005–2006)	Nationally representative survey of 7607 civilian, non‐institutionalized subjects, aged ≥ 20 years. N in Table 2 total 7707 patients, therefore, percentages differ from Crook et al, 2021. There are 100 extra patients reported in the Adequate group.	Population (national)	54.4 (28.6)	6.1	9.5	26.2	33.7% (2567/7607)	25.8	[3]
United States	Crook et al.	2023	Cross‐sectional study. Secondary analysis of NHANES survey (2003–2004, 2005–2006)	7607 civilian, non‐institutionalized participants. ≥ 20 years.	Population (national)	54.63 (28.62)	Total 6.1; M/F 8.3/4.1	Total 9.5; M/F 11.5/7.6	Total 26.2; M/F 28.7/23.8	Total 32.4; M/F 32.2/32.7	Total 25.8 M/F 19.3/31.9	[12]
United States	Dionne et al.	2016	Cross‐sectional study. Secondary analysis of NHANES survey (2003–2004)	4742 civilian, non‐institutionalized subjects, aged ≥ 20 years. 304 had missing values for Vitamin C levels.	Population (national)	Nil data	8.4 [< 12]	9.4 [12–23.9]	28.2 [24–52.9]	27.8 [53–70]	26.1	[38]
United States	Dionne et al.	2018	Cross‐sectional study. Secondary analysis of NHANES survey (2003–2004)	Nationally representative survey of 4438 individuals, aged ≥ 20 years.	Population (national)	Nil data	8.4	9.4	28.2 [23.1–52]	27.8 [52.1–70].	26.1	[21]
United States	Driskell et al.	2006	Cross‐sectional study	22 children, 2–5 years, from 3 daycare centres. 13 males, 9 females of mixed ethnicity. Nil illnesses or regular medications, 7 children took supplements containing vitamin C.	Daycare centres (Community)	M 24 (9.8) F 29 (7.2)	Total 32; 2–3 years 40		68 [≥ 23]			[46]
United States	Ebenuwa et al.	2023	Cross‐sectional cohort study	96 women, 56 without HIV (PWOH), 40 with HIV (PWH). 18–65 years. In community participants, acute or chronic illness, alcohol abuse, smoking and chronic medications were excluded.	National Institutes of health Clinical Center and Georgetown University (outpatient and community)	PWOH 50 (23) PWH 14 (12)	PWH 43, PWH 7	Nil data	Nil data	Nil data	Nil data	[45]
United States	Gordon et al.	2022	Retrospective cohort study	301 patients, ≥ 18 years with diagnosis of Crohn's disease (CD) or Ulcerative Colitis (UC) and plasma vitamin C levels.	Inflammatory bowel disease clinic (outpatient)	35.7 (27.8)	Total 21.6; CD 24.4, UC	Total 24.6	Total 53.8			[4]
United States	Hampl et al.	2004	Cross‐sectional study. secondary analysis of nhanes survey (NHANES III)	15,769 nationally representative survey of civilian, non‐institutionalised children and adults, 12–74 years.	Population (national)	12–17 yrs M/F 46 (1.7)/50 (1.7) 18–24 yrs M/F 36.9 (1.1)/43.7 (1.7) 25–44 yrs M/F 36.3 (1.1/42.6 (1.1) 45–64 yrs M/F 38 (1.1)/47.7 (1.1) 65–74 yrs M/F 44.9 (1.1)/55.1 (1.1)	M/F 14/10 12–17 yrs M/F 6/5, 18–24 yrs M/F 13/11, 25–44 M/F 17/12 45–64 M/F 17/10, 65–74 M/F 14/6.	M/F 20/17. 12–17 yrs M/F 15/17, 18–24 yrs M/F 22/19, 25–44 yrs M/F 23/20, 45–64 yrs M/F 20/15, 65–74 yrs M/F 15/13	M/F 66/73, 12–17 yrs M/F 77/80, 18–24 yrs M/F 65/70, 25–44 60/68, 45–64 M/F 63/75, 65–74 M/F 74/81.			[39]
United States	Kimmons et al.	2006	Cross‐sectional study. Secondary analysis of NHANES survey (NHANES III)	16,191 participants of a nationally representative survey of civilian, non‐institutionalised adults, aged 19 years and older. Pregnant and lactating women, BMI < 18.5 were excluded	Population (national)	Nil data	Premenopausal women: Overweight 20.02, Obese 1.13; Men overweight 29.89, Obese 1.25 [< 22.7]			Nil data	Nil data	[11]
United States	Mangano et al.	2021	Cross‐sectional Study. Secondary analysis from Boston Peurto Rican Health Study.	902 Puerto Rican adults living in US. 809 had samples for vitamin C levels collected.	Population cohort (community)	Means ranged from 44.4 (23.5) to 54.9 (20.1) ≥ 50 µmol/L across dietary intake tertiles	56			44		[13]
United States	Pfeiffer et al.	2013	Review of the Second Nutrition Report, based on NHANES data.	Sample of over 14,579 participants from NHANES 2003–2006. ≥ 1 year old.	Population (national)	Nil data	6	Nil data	Nil data	Nil data	Nil data	[40]
United States	Powers et al.	2023	Cross‐sectional study. Secondary comparitive analysis of NHANES survey data 2003–2018	13,824 participants, ≥ 20 years with serum vitamin measures.	Population (national)	M/F 46.1/56	2017–2018 Total 6.8	Nil data	Nil data	Nil data	Nil data	[41]
United States	Salo et al.	2022	Cross‐sectional Study. Pooled analysis NHANES 1988–2006	Nationally representative survey of 34,056 individuals, aged ≥ 20 years.	Population (national)	Nil data [median 42.02]	NHANES III 15.1, Continuous NHANES 7.0	Nil data	Nil data	Nil data	Nil data	[42]
United States	Schleicher et al.	2009	Cross‐sectional Study. NHANES 2003–2004	7277 civilian, non‐institutionlized children and adults. ≥ 6 years.	Population (national)	51.4	Low income 17.4, high income 7.9, ≥ 6–7.1, ≥ 20 years 8.4	≥ 6 yrs 19.6 (1.9), ≥ 20 yrs 22.2 (2.1) [total < 28]	≥ 6 80.4, ≥ 20 yrs 77.8 [> 28]			[43]
United States	Sun et al.	2022	Retrospective cohort study	Review of 52,150 mortality records of adults, 18–85 yrs of the 1999–2018 NHANES survey. Death due to accident and records lacking mortality data were excluded. Absence of death record were considered alive.	Population (national)	Normal weight 52.81, Obese class III 40.88	Non‐diabetic M/F 8/5.5, T1DM M/F 1/6, T2DM M/F 8/7.5	Nil data	Nil data	Nil data	Nil data	[35]
United States	Tobon et al.	2008	Cross‐sectional study	Nutritional status of 8 English‐speaking patients, aged 21–100 years. with chronic leg ulcers were assessed. Excluded if infection or ulcers of nonvenous cause.	Wound clinic (outpatient)	47.13	25 [< 17]	12.5 [17–26.12]	62.5 [> 26.12]			[34]
United States	Zheng et al.	2022	Cross‐sectional Study (NHANES 2003–2006) data	2174 women, aged 18–59 years.	Population ‐ women (national)	Nil data	14.8 [< 24]		25 [24–49.99]	32.6	27.6	[44]
Canada	Cahill et al.	2009	Cross‐sectional study	979 adults, aged 20–29 years. Participants of the Toronto Nutrigenomics and Health Study from 2004–2008. Smokers, inaccurate caloric intake reporting, pregnancy and breastfeeding excluded (adults ‐ young)	University students (community)	Crude 30.6 (0.6), Adjused 27.2 (0.9) M/F 24.4/30	14	33	53 [> 28]			[48]
Canada	Langlois et al.	2016	Cross‐sectional study (Secondary analysis of Canadian Health Measures Survey)	1615 adults, aged 20–79 years, as subset of CHMS, which is representative of 96% of Canadians. Military personnel, residents of institutions, Aboriginal settlements and some remote regions are not included.	Population (national)	53	Total < 3 Never consume citrus 13, Rarely consume fruit juice 100, Smokers 10	Nil data	Nil data	Nil data	Nil data	[47]
Canada	Zeitoun & El‐Sohemy	2023	Cross‐sectional Study	555 20–29 year ol female participants from Toronto Nutrigenomics and Health study.	Population (community)	Deficient 6.2 (3.2), inadequate 20.3 (4.8), adequate 44.4 (13.4)	10	26	64 [> 28]			[49]
Mexico	Garcia et al.	2012	Cross‐sectional study	580 adult women, 25–55 years. Pregnancy, lactation and uncontrolled diabetes or hypertension were excluded. 513 women had vitamin C blood samples collected.	Rural communities (community)	Total 12.49. Normal weight 30.66 (12.49), BMI > 25 30.66 (12.49) BMI> 30 28.96 (12.49)	Total 5 normal weight 2.9, BMI > 25 5.7, BMI > 30 5.7.	Total 32.4 Normal weight 29.6, BMI > 25 28.4, BMI > 30 36.9% [11.36–22.7]	Total 62.6 [≥ 22.7]			[75]
Mexico	Garcia et al.	2013	Cross‐sectional study	197 children, 6–10.5 years from 2 rural schools. Supplementation in past month, Type I diabetes and physical or mental disability excluded.	Rural schools (school)	22.71 (8.58)	Nil data	38.1 [22.7]	61.9 [≥ 22.7]			[76]
Mexico	Rivera & Amor	2003	Cross‐sectional study, data from Mexican National Nutrition Survey 1999.	8011 children < 5 years, 11,415 children 5–11 years, 18,311 women 12–49 years were surveyed and tested as a representative population sample of national, regional, urban and rural segments of Mexico.	Population (national)	Nil data	1–2 years 30, 3–4 years 25, 5–11 years 30, Women 40 [11.36]	Nil data	Nil data	Nil data	Nil data	[72]
Mexico	Villalpando et al.	2006	Cross‐sectional study, data from Mexican National Nutrition Survey 1999.	1770 children, aged 0.5–11 years, from households randomly selected to participate in study.	Population (national)	Anaemic 8.97 (7.15), Non‐anemic 8.97 (5.39)	32.4 [< 17.03]		67.6 [≥ 17.03]			[74]
Mexico	Villalpando et al.	2003	Cross‐sectional study, data from Mexican National Nutrition Survey 1999.	1966 children, aged 0.5–11 years, and 960 non‐pregnant women, aged 12–49 years).	Population (national)	Non‐pregnant women 19.31, Children 28.39	Women 39.3 Children 23, 0–2 yrs 30.3, 3–4 yrs 24.2, 5–6 yrs 17.3, 7–8 yrs 21.5, 9–10 yrs 19.8, 11 yrs 31.1. Total for children 23 [11.36]	Total 25 [≤ 17.03]	Total 75 [> 17.03]			[73]
Cuba	Lanyau‐Dominguez et al.	2020	Cross‐sectional study. Sub‐sample from the aging and Alzehimer study.	424 participants. ≥ 65 years. 43 with Alzheimer's disease (AD), 131 with mild cognitive impairment and 250 with no signs of cognitive impairment (CI), from 4 areas of Havana. Severe medical or psychiatric conditions excluded.	Population 65+ (community)	No impairment 79.1 (64) Mild CI 75.5 (45.6), AD 61.6 (51.9)	No impairment 4, CI 4.1, AD 21.1.	Nil data	Nil data	Nil data	Nil data	[18]
South America												
Brazil	Arruda et al.	2013	Randomized, double‐blind, placebo‐controlled trial	83 adult patients with sickle cell anaemia and thalassaemia. 18–68 years. 64% female.	Hereditary anaemia outpatient clinic (outpatient)	Baseline 27.2, supplements 62.6	Baseline 30	Baseline 30 [11–29.9]	Baseline 40 [30–150]			[79]
Brazil	Coelho et al.	2020	Cross‐sectional observational study	72 patients. > 19 years (28–81) with non‐alcoholic fatty liver disease (NAFLD). 77.8% female. Pregnancy, lactation, smoking, infectious diseases, renal failure, liver transplant, cancer or certain medications or chemotherapy or supplement use in previous 6 months were excluded.	Outpatient hepatology clinic (outpatient)	Nil data (median 32.48)	27 [< 26.12]		73 [≥ 26.12]			[80]
Brazil	Hermes Sales et al.	2023	Cross‐sectional study (secondary analysis from 2015 ISA‐Nutrition survey).	824 participants. ≥ 14 years. Household residents of Sao Paulo. Exclusion criteria included enteral/parenteral nutrition, pregnant or lactating women, alcohol dependence.	Population (community)	14–18 yrs M/F 38.5/34.8 19–30 yrs M/F 39.3/39.8 31–50 yrs M/F 35.7/40.8 51–70 yrs M/F 42.3/58 ≥ 71 yrs M/F 48.5/55.4	14–18 yrs M/F 23.7/31 19–30 yrs M/F 23.5/21.6 31–50 yrs M/F 22.9/11.4 51–70 yrs M/F 13/10.6 ≥ 7 1yrs M/F 10.9/7.5	Nil data	Nil data	Nil data	Nil data	[77]
Brazil	Higa Junior et al.	2017	Cross‐sectional Study	66 participants (60.6% males), ≥ 18 years, without intellectual disability and who worked as a waster picker in Campo Grande.	Waste picker cooperatives and landfill sites (community)	Nil data	98.5% [< 26.13]		1.5 [≥ 26.13]			[81]
Brazil	Machado et al.	2013	Cross‐sectional Study	49 pregnant women with HIV. ≥ 14 years. No multivitamin supplements,	Antenatal clinic (outpatient)	Nil data [median 43.8]	0%	12.2	87.8 [≥ 28]			[82]
Brazil	Stenzel et al.	2018	Cross‐sectional study	60 obese adolescents, 10–20 years. Excluded if weight‐loss surgery, malabsorption, cancer, treatments for cholesterol of diabetes, any supplements, pregnancy and lactation, liver or kidney diseases.	Obesity clinic (outpatient)	8.52 (3.41)	91.7 [< 26]		Nil data	Nil data	Nil data	[78]
Ecuador	Hamer et al.	2009	Cross‐sectional study	352 participants (225 women and 127 men), ≥ 65 years, mentally competent and of low socioeconomic status from 3 neighbourhoods.	Periurban communities 65+ years (community)	M/F 11.4 (9.1)/17 (10.2)	M/F 59.8/32.6	Nil data	Nil data	Nil data	Nil data	[84]
Ecuador	Sempertegui et al.	2011	Cross‐sectional study	352 participants (225 women and 127 men), aged 65 years and over, mentally competent and of low socioeconomic status from 3 neighbourhoods.	Slum (community)	Nil data (median 10th percentile 9.65, 90th percentile 14.76)	82	Nil data	Nil data	Nil data	Nil data	[83]
Venezuela	Ruiz‐Fernandez et al.	2016	Cross‐sectional study	114 children from 6 public schools. 7‐9 years belonging to SES of middle‐class or critical poverty. Healthy, with no medical illnesses.	School (school)	55.64 (24.42)	Total 47.4, middle class 34.4, critical poverty 62.3 [< 51.1]			52.6 [≥ 51.1]		[85]
Europe												
Germany	Back et al.	2004	Cross‐sectional study	22 patients with Cystic Fibrosis, 35 healthy controls from staff and endocrinology clinic. Pregnancy, smokers, breastfeeding, acute infections and severe liver disease excluded.	Outpatient clinics (outpatient)	6–11 yrs CF/Control 68.4/65.4, 12–17 yrs CF/Control 53.3/65.3, ≥ 18 yrs CF/Control 47.3/64.3	CF 0, controls 0	6–11 yrs CF/Control 0/11, 12–17 yrs 43/0, ≥ 18 yrs CF/Controls 80/6 [11–50]		6–11 yrs 100/89, 12–17 yrs CF/Controls 57/100, ≥ 18 yrs CF/Control 20/94 [> 50]		[50]
Germany	Damms‐Machado et al.	2012	Randomised control trial. Pilot study	104 obese participants in OPTIFAST weight loss program, randomly allocated to different subgroups for low calorie diet interventions. History of bariatric surgery excluded. Median age 45.8 (11) years.	Weight loss program (community)	Baseline 59.44 (22.73) after 37.29 (13.48)	> 15 [28.3]		Nil data	Nil data	Nil data	[53]
Germany	Hagel et al.	2018	Cross‐sectional study	188 (138 female, 50 male) volunteers attending a food allergies and intolerance seminar. Aged 21–86 years.	Food allergy and intolerance seminar (community)	44	3.3 [< 8.52]	14.1 [< 28.39]	20 [28.39–39.75]	63 [> 40]		[51]
Germany	Jungert & Neuhauser‐Berthold	2015	Cross‐sectional study	270 participants (181 females, 89 males) in final analysis of physically mobile, independently living adults in Giessen. ≥ 60 years.	Population cohort 60+ (community)	Nil data median M/F 61.9/76.1]	0.7 [< 28]		11.4 [28–49.99]	87.9 [> 50]		[52]
United Kingdom	Elia & Stratton	2005	Cross‐sectional Study. Secondary analysis of data from National Diet and Nutrition Survey.	1155 participants ≥ 65 years, free‐living and institutionalised in the community from 80 areas within the UK. Blood samples from 1276, 1012 had vitamin C levels.	Population cohort 65+ years (national)	Northern 29.67 (1.29), Central 38.08 (1.32), Southern 45.52 (1.08)	North/central/southern Severe deficiency (< 5) 15/5.2/2.1. Mild deficiency (< 11) 33/20/10	Nil data	Nil data	Nil data	Nil data	[54]
United Kingdom	Finck et al.	2015	Cross‐sectional study and prospective study	Total of 3833 participants included in Hip fracture and Ultrasound with vitamin C levels per quintile, as a sub‐sample of the larger EPIC‐Norfolk study. 39–70 yrs.	Population (national)	Ultrasound study (*n* = 2327): M/F 48.7 (17)/60.3 (19.9) Fracture study (*n* = 4510): M/F 46.4 (18.2)/58.2 (20.1)	Males 20.8 [3–31], 20.5 [32–43], Females 20.7 [4–43]. ~Hip fracture groups		Males 20.8 [44–52], 20.9 [44–55]~	Males 18.4 [53–61], Females 18.5 [56–63], 20.8 [64–73]~	Males 19.5 [62–132], Females 19.1 [74–170]~	[55]
United Kingdom	Gosney et al.	2008	Double‐blinded randomised controlled trial	73 nursing and residental home residents, ≥ 60 years from 11 facilities. No antidepressants for 2 weeks or during assessment period with normal thyroid function. Micronutrient supplement containing 120 mg vitamin C vs. placebo.	Nursing homes, population cohort 60+ years (community)	Baseline 20.15, after supplementation 64.1	Baseline 67, After supplementation 1.8 [< 23]		Baseline 33, After supplementation 98.2 [23–118]			[60]
United Kingdom	Lewis et al.	2020	Cross‐sectional Study. Secondary analysis of data from UK EPIC‐Norfolk study	Analysis of 5853 men and 7212 women with plasma vitamin C level conducted as part of the original study group. 40–79 years.	Population (national)	M/F 56.9 (21.3)/68.9 (24.5)	M/F 0.9/0.2 women	M/F 34.7/16.8		M/F 66.4/83 [≥ 50]		[56]
United Kingdom	MacMaster et al.	2021	Cross‐sectional study	93 patients with Inflammatory Bowel Disease. 19–79 years.	Tertiary referral unit, (outpatient)	Nil data	13 (nil definition provided)	Nil data	Nil data	Nil data	Nil data	[61]
United Kingdom	McCall et al.	2019	Cross‐sectional study	22,474 participants from a population‐wide study EPIC‐Norfolk, had blood levels for vitamin C performed. 40–79 years.	Population 40–79 years (national)	Nil data	1.4	10.70%	87.9 [> 28]			[57]
United Kingdom	McGrogan et al.	2021	Cross‐sectional and longitudinal study	78 Children attending hospital in Glasgow for routine follow up of Coeliac Disease or suspected Coeliac disease. Median age 9.3 (3.5).	Gastrointestinal outpatient clinic (outpatient)	Nil data	Low level 2.2 (nil reference provided). Batch failure of samples provided 46 samples only, of which 1 was low.	Nil data	Nil data	Nil data	Nil data	[59]
United Kingdom	Mosdol et al.	2008	Cross‐sectional Study (secondary analysis of the low‐income diet and nutrition survey)	433 men and 876 women from the lowest‐income households in the UK, aged 19 years or older. Pregnant women excluded. Non‐fasting samples.	Population (national)	Nil data	M/F 25.3/16.1	M/F 21/18.5	M/F 53.7/65.4 [> 28]			[58]
Austria	Fabian et al.	2011	Cross‐sectional study	102 healthy, independent, non‐institutionilzed subjects. 70–90 years, representative of state of Burgenland.	Population cohort 70–90 years (community)	Total 70 (22) ≤ 2 meds per day 72 (22) 3 meds per day 68(21)	0 [< 20]	9 [20–37]	91 [> 37]			[62]
Austria	Fabian et al.	2012	Cross‐sectional study	102 healthy, independent, non‐institutionilzed subjects. 70–90 years, representative of state of Burgenland. 2% took supplements.	Population cohort 70–90 years (community)	Total 70 (22) supplemented 80 (18) unsupplemented 60(20)	0 [< 20]	9 [20–37]	91 [> 37]			[63]
Poland	Godala et al.	2016	Case‐control study	182 patients with Metabolic Syndrome (MS) aged 30–65 years, and 91 healthy control participants aged 41–65 years. Non‐smokers and nil supplements in past year.	Clinic (outpatient and community)	MS 31.18 (9.21) Controls 58.43 (18.15)	Nil data	Cases 79.12 Controls 8.79 [< 36.1]	Cases 20.88 Controls 91.21 [≥ 36.1]			[127]
Poland	Szczuko et al.	2014	Cross‐sectional study	120 university students (96 women, 24 men). 24.6% had taken vitamin C supplements in the 7 days before samples.	University (community)	M/F 45.61/48.65	0.8	6.7	18.3 [29–39.9]	49.1 [40–55]	25 [> 55]	[64]
Denmark	Juhl et al.	2017	Cross‐sectional study	47 pregnant women with Type 1 Diabetes, > 18 years with no other diseases, singleton pregnancy.	Obstetric clinic (outpatient)	30.1 (13.6)	28 [< 23]		72 [≥ 23]			[65]
Slovakia	Krajcovicova‐Kudlackova et al.	2007	Cross‐sectional study	368 healthy adults randomly selected from Bratislava who consumed either traditional mixed diet or vegetarian diet. 19–68 years.	Population (community)	Vegetarians 75.8 (1.2) Mixed diet 53.0 (0.9)	Total 11.23 [< 23]		Total 42.25 [23–50]	Total 46.52 88 Mixed diet 46 [> 50]		[66]
The Netherlands	Kuzmanova et al.	2012	Case‐control study	21 patients ≥ 21 years with periodontitis. 21 controls matched for age, gender, race and smoking.	Dental clinic (health care service)	Cases 47.13 (22.14) Control 64.16 (29.53)	Case 0 Controls 0 [< 11.36]	Cases 19 ‐ nil data [11.36–22.14]	Cases 81, Control ‐ nil data [> 22.14]			[67]
The Netherlands	Sotomayor et al.	2017	Prospective cohort study	598 renal transplant recipient patients, beyond 1 year since transplant with no current illness. Mean age 51 (12) years.	Renal transplant clinic (outpatient)	Nil data [median 44]	22 [≤ 28]		78 [< 28]			[68]
France	Filippi et al.	2006	Case‐control study	54 patients with Crohn's Disease, 25 healthy controls, 18–70 years. Excluded comorbid conditions likely to affect nutrition, glucocorticoids in past 2 months and enteral or parenteral nutrition in the past year.	Gastrointestinal outpatient clinic (outpatient clinic and community)	Nil data	CD 84 (no reference range provided) Control ‐ nil data		Nil data	Nil data	Nil data	[69]
Switzerland	Schupbach et al.	2017	Cross‐sectional study	206 volunteer subjects recruited through advertising campaign by Swiss Vitamin Institute and ETH Zurich. Following either vegan (VN), omninorous (OV) or vegetarian (VG) diet for at least 12 months. 18–50 years.	Community (community)	OV 54.3 (17.2) 68.9 (23.37) VN 71.8 (23.4)	OV 12, VG 3.8, VN 3.8 [< 35.4]		Nil data	Nil data	Nil data	[70]
Africa												
Nigeria	Ene‐Obong et al.	2003	Cross‐sectional study	600 in‐school adolescents, 10–20 years. 45 participants randomly selected for vitamin C levels.	Schools in Enugu State region (school)	13–15 yrs M/F 42.02 (9.65)/50.53 (17.6) 16–18 yrs M/F 43.15 (12.49)/46.56 (23.28) 19–20 yrs M/F 34.64 (14.2)/49.97 (8.52)	46.7 [< 39.75]		48.9 [39.75–79.49]		4.4 [> 79.49]	[98]
Nigeria	Odum & Wakwe	2012	Case‐control study	200 patients attending a metabolic syndrome clinic, 200 hospital staff. Acute or chronic illness, supplementation, pregnancy, alcoholism, smoking were criteria for exclusion. Median age 51.44 (10.88) years.	Outpatient clinics (outpatient/community)	Cases 29.05 (7.32), Controls 43.94 (7.26)	Cases 0, Controls 0 [< 16.5]	Cases 32 0 [16.5–< 23]	Cases 68, Controls 100 [≥ 23]			[97]
Nigeria	Olayiwola et al.	2014	Cross‐sectional study	120 free‐living participants. ≥ 60 years from Yoruba region. Serious illess, immobility and institutionization excluded.	Population 60+ years (community)	Total 50, M/F 60/40 [< 31.23]			Total 50, M/F 40/60 [≥ 31.23]			[99]
Nigeria	Onyesom & Osioh	2011	Case‐control study	50 newly diagnosed Type 2 diabetics, 50 controls from hospital community. 45.37 (9.7) years.	Diabetes clinic (outpatient/community)	Cases 37.47 (9.65), Control 55.08 (13.06)	Nil data	Cases 36, Controls 0 [< 28.39]	Cases 20, Controls 5 [28.39–39.18]	M/F 20/70 [> 45.42]		[102]
Nigeria	Opara et al.	2007	Randomised control trial	290 patients. 18–69 yrs with HIV, attending clinic for dispensing of anti‐retroviral therapy. 144 received nutritional counselling and free micronutrients (NS), 146 received neither (control).	HIV clinic (outpatient)	Baseline 17.03–51.1	Baseline NS 60–100, baseline controls 70–100, after control 86 [< 28.39]		After NS 100, after control 14 [≥ 28.39]			[103]
Nigeria	Ugwa et al.	2016	Prospective cohort study	400 pregnant women attending antenatal clinic at General Hospital Dawakin Kudu. Childbearing age.	Antenatal clinic (outpatient)	19.87 (28.96)	79.5 [< 28.39]		20.5 [28.29–113.56]		0 [> 113.56]	[101]
Nigeria	Ugwa et al.	2015	Prospective cohort study	216 pregnant women attending antenatal clinic at General Hospital Dawakin Kudu. Hypertension, diabetes, other illnesses excluded. Childbearing age.	Antenatal clinic (outpatient)	19.87 (29.53)	79.5 [< 28.39]		20.5 [28.29–113.56]		0 [> 113.56]	[100]
Uganda	Kiondo et al.	2012	Case‐control study	215 pregnant women with pre‐eclampsia at least 20 wks pregnant, 400 pregnant women with normal pregnancy, 200 non‐pregnant women. Severe medical conditions excluded. 15–49 years, living within 15 km of the hospital.	Antenatal and family planning clinics (outpatient)	Pre‐eclampsia 9.77 (3.86), normal 10.73 (4.14), non‐pregnant 14.99 (5.51)	Total 58.6%, pre‐eclampsia 70.2, normal 68, non‐pregnant 25 [< 11.36]	Pre‐eclampsia 28.9, normal 31.5, non‐pregnant 63.5 [11.36–22.7]	Pre‐eclampsia 0.9, normal 0.5, non‐pregnant 11.5 [> 22.71]			[119]
The Gambia	Moore et al.	2003	Cross‐sectional and interventional study	472 children from 22 villages in rural Keneba, participating in a vaccination efficacy trial. 6.5–9.5 years.	Rural villages (community)	50 (32.2)	9	Nil data	Nil data	Nil data	Nil data	[120]
Central African Republic	Moya‐Alvarez et al.	2021	Prospective cohort study	48 mothers, 50 infants from Bangui were monitored for 6 months from birth as part of the MITICA (Mother‐to‐Infant Transmission of microbiota in Central‐Africa) study. Mothers 15–39 years. Mothers excluded if positive for HIV, HBV or HCV.	Urban (outpatient)	Nil data [median women at birth 15.3/Infants at birth 35.2]	Women at birth 42.2 Infants at birth 15 Infants at 11 wk 24.3 Infants at 25 wk 33.3	Women at birth 33.4 Infants at birth 27.5 Infants 11 wk 10.8 25 wk 21.3	Women at birth 24.4 infants at birth 57.5 infants 11 wk 64.9 infants 25 wk 45.4			[121]
Asia												
India	Agrawal et al.	2003	Prospective cohort study	100 patients with non‐healing wounds, 10–78 years. 85 males, 15 females. 41 patient had vitamin C levels collected.	Wound Clinic, outpatient department (outpatient)	Cases 38.04 (19.31), Controls 79.49 (19.31)		Cases 41.5 [< 28.39]		Cases 51.2 [28.39–56.78]	Cases 7.3 [> 56.78]	[111]
India	Augustine et al.	2012	Cross‐sectional study	109 students. 15–19 years, male and females, students from low and middle‐income families.	Government‐run secondary school (school)	23.85 (16.47)	Nil data	75% in subset of boys (nil reference provided)	Nil data	Nil data	Nil data	[108]
India	Bansal et al.	2014	Cross‐sectional study	775 adolescent girls, 11–18 years.	Slum (community)	43.15 (25.55)L	6.3 [< 11.36]	27.6 [11.36–22.71]	66.1 [> 28.39]			[105]
India	Chiplonkar & Kawade	2012	Randomized control trial	180 girls. 10–16 years. Recent illness, current medical treatment or supplement intake excluded.	Secondary school (school)	Total baseline 18.74 (0.57)	Baseline (pooled) 15.3 [< 11.36]	Baseline 66.7 [11.36–22.71]	18 [> 22.71]			[109]
India	Kawade	2012	Cross‐sectional study	630 girls, aged 10–16 years from 2 secondary schools in Pune City. Exclusion criteria included any illness, medical treatment or multivitamin supplements.	Secondary school (school)	Nil data	10.8 [< 11.36]	67.6 (“low plasma vitamin C,” nil reference provided)	Nil data	Nil data	Nil data	[106]
India	Kehoe et al.	2015	Randomised control trial (adjunct to Mumbai Maternal Nutrition Project)	222 non‐pregnant non‐lactating women, aged 14–35 years living in a slum in Mumbai. Interventional trial assessing response to 12 weeks of supplementation. Treatment snack contained 2.1 mg vitamin C, control nil.	Slum (community)	Nil data [baseline median control/treatment 12/12.7. After 12 weeks 14.6/17]	40	60 [> 11]				[110]
India	Ravindran et al.	2011	Cross‐sectional study	7518 participants randomly sampled from northern and southern India. 5638 people ≥ 60 years, included in vitamin C analysis. Assessments performed for cataract, blood parametres and questionnaire.	Population cohort ≥ 60 years (national)	Nil data	25 [< 2], 25 [3.1–5.7]	25 [9.9–16.2]	25 [28.2–52.5]	0	0	[9]
India	Ravindran et al.	2011	Cross‐sectional study	7518 participants randomly sampled from northern and southern India. 5638 people ≥ 60 years, included in vitamin C analysis. Assessments performed for cataract, blood parametres and questionnaire.	Population cohort ≥ 60 years (national)	Nil data	North India 73.9 South India 47.5	North India 15.3 South India 28.4	North India 10.8 South India 25.9 [> 28]	0	0	[77]
India	Sivakumar et al.	2006	Randomized double‐blind placebo‐controled trial	Number of participants not provided. School children, aged 6–18 years. Supplement vs. placebo.	School (school)	Baseline supplement/Placebo 33/30. After 14 months supplement/Placebo 61/54	Total baseline 59.6 [< 30]		Total baseline 40.4 [≥ 30]			[104]
India	Thankachan et al.	2013	Randomised, double‐blind placebo‐controlled trial	238 School children who had vitamin C levels taken. 6–12 years, with serum ferritin levels < 20 µg/L. Excluded if severe anaemia, chronic illness or dsability or severe malnutrition. Multiple micronutrient (MMN) drink vs. placebo. 27 mg of vitamin C in MMN drink.	School (school)	Baseline MMN/Placebo 28.96 (16.47)/26.12/15.9	Baseline MMN/Placebo 37/47 After 8 wks MMN/Placebo 16/42 [≤ 22.71]		Baseline MMN/Placebo 63/53 after 8 wks 84/58 [> 22.71]			[107]
Bangladesh	Ahmed et al.	2005	Randomized double‐blind control trial	197 anaemic girls (Hb < 12.9g/dL), 14–18 years, postmenarchal, non‐pregnant. Severe anemia/chronic infection/metabolic disorder excluded. Iron Folic Acid (IFA) supplements vs. Multiple Micronutrient (MMN)	Rural schools (school)	Baseline IFA/MMN 42.59 (1.7)/43.72 (2.27)	Baseline IFA/MMN 5.6/6.7 After IFA/MMN 10.1/0 [16.47]	Nil data	Nil data	Nil data	Nil data	[112]
Bangladesh	Ahmed et al.	2008	Cross‐sectional study	310 anaemic, post‐menarchal school girls. 14–18 years. 307 participants had plasma Vit C levels collected.	Rural schools (school)	46.1 (20)	2	9.6 [11.4–23]	88.4 [≥ 23]			[113]
Bangladesh	Ahmed et al.	2010	Randomized double‐blind control trial	324 anaemic (Hb < 120g/L) post‐menarchal high‐school girls, 11–17 yrs. Chronic diarrhoea/severe anemia/regular medication excluded. Iron Folic Acid (IFA), Multiple Micronutrient supplement twice weekly (MMN2), Multiple Micronutrient once weekly (MMN1).	Rural schools (school)	Baseline IFA/MMN2/MMN1 24.4 (14.4)/27.2 (13.8)/26.6 (14.5) After 26wks IFA/MMN2/MMN1 27.6 (16.3)/37.7 (13)/35 (12.8) After 52wk IFA/MMN2/MMN1 25.7 (13.4)/36.8 (13.3)/34.1 (13.3)	Baseline IFA/MMN2/MMN1 29.1/29.4/30 26wk IFA/MMN2/MMN1 33.8/1.5/3.8 52wk IFA/MMN2/MMN1 31.1/0/3.8 [< 16.5]	Nil data	Nil data	Nil data	Nil data	[114]
Dhaka, Narayanganj and Narshingdi Districts, Bangladesh	Ahmed et al.	2012	Randomized double‐blind control trial	324 non‐anaemic (Hb> 120g/L), non‐pregnant, post‐menarchal high‐school girls, 11–17 yrs. Any disease/prior supplementation excluded. Iron Folic Acid (IFA), Multiple Micronutrient supplement twice weekly (MMN2), Multiple Micronutrient once weekly (MMN1).	School (school)	Baseline IFA/MMN2/MMN1 21.1/22/23.5 after 26 wk IFA/MMN2/MMN1 24.3/34.1/31.1 after 52 wk 23/34.3/31.4.	Baseline IFA/MMN2/MMN1 28.4/27.1/31.9 After 26 wk 19.4/4.3/5.6 After 52 wk 25.4/0/5.6 [< 16.5]	Nil data	Nil data	Nil data	Nil data	[115]
Bangladesh	Kabir et al.	2010	Cross‐sectional study	65 female school student, 15–19 years from Home Economic college. Excluded if any signs of disease.	School (school)	68.14 (34.07)	8 [< 16.47]	92 [≥ 16.47]				[116]
Sri Lanka	Allen et al.	2021	Cross‐sectional study	162 patients with ß‐thalassemia. Children, adolescents and adults. 62.3% females, 37.7% males. Median age 26 (IQR 15.3–38.8)	Outpatient thalassemia care services (outpatient)	Nil data	82.5 [< 14]	Nil data	Nil data	Nil data	Nil data	[117]
Indonsesia	Amaliya et al.	2007	Longitudinal study	123 subset of original 128 participants of Java project in 2002. 64 females, 59 males. 33–43 years.	Village inhabitants on Malabar/Purbasari tea estate (community)	44.86 (30.38)	14.7 [< 11.36]	13.8 [11.36–22.14]	71.5 [> 22.14]			[14]
Indonsesia	Amaliya et al.	2015	Longitudinal study	98 participants, 39–50 years. Participants selected from previous study population described by Amaliya et al (2007).	Purbasari tea estate (community)	Total 29.47 (18.74), normal 41.68 (16.86), depleted 16.35 (3.58), deficient 8.4 (1.93)	11.2 [< 11.36]	33.7 [11.36–22.14]	55.1 [> 22.14]			[118]
Thailand	Assantachai & Lekkakula	2007	Cross‐sectional study	2336 participants, aged 60 years and over from 4 regions of Thailand. Males:females 1:1.63.	Population cohort 60+ years (national)	Nil data	9.9 [< 22.71]		90.1 [> 22.71–85.17]			[86]
Thailand	Boonpangrak et al.	2018	Cross‐sectional study	250 participants from 10 workplaces (3 construction and 7 business offices). 32–43 years. Chronic disease, regular medication, vitamin C supplementation, pregnant or breastfeeding excluded. Divided into 5 groups based on behaviours. 1 = office worker, non‐smoker, non‐drinker. 2 = drinker. 3 = outdoor worker. 4 = smoker. 5 = outdoor worker, smoker and drinker.	Construction and office workplaces (community)	Nil data	Group 1/2/3/4/5. %8/10/24/22/44	Group 1/2/3/4/5. %16/22/18/38/26 [11–22]	Group 1/2/3/4/5. %76/68/58/40/30 [23–85]			[88]
Thailand	Siripornpanich et al.	2022	Cross‐sectional study	53 male, 23 female asthmatic children and adolescents attending the allergy clinic at Ramathibodi Hospital. 7–17 years. Extensive exclusion criteria applied.	Allergy clinic (outpatient)	Mild/Moderate/Severe 19.25 (11.87)/19.93 (11.07)/11.19 (10.84)	39.5 [< 11.36]	60.5 [11.36–113.56]				[87]
Thailand	Tungtrongchitr et al.	2003	Cross‐sectional study	149 healthy overweight and obese (BMI≥ 25), 113 healthy normal weight (BMI< 25) participants, 18–60 years. Females overrepresented.	General practice (health care service)	Nil data [median overweight 19.87, normal weight 36.91]	Total BMI≥ 25 62.7 [< 28.39]		Total BMI≥ 25 38.3 [≥ 28.39]			[89]
Taiwan	Chen et al.	2011	Case‐control study	50 healthy controls from hospital‐based volunteer pool, 50 patients with Post Herpetic Neuralgia (PHN). Admission or infection within 6 months, chronic pain above 3 on scale or regular supplement use excluded. Average 63.6 years.	Pain clinic and healthy hospital volunteers (outpatient)	PHN 30 (20.8), Controls 76.2 (31.2)	PHN 0, Controls 0	PHN 52, Controls 0 [11.3–22.6]	PHN 48, Controls 10 [26.1–84.6]			[90]
Taiwan	Lin et al.	2022	Retrospective cross‐sectional study	3899 individuals ≥ 20 years, with serum Vitamin C checked as part of periodic health examinations under national health insurance. HIV, diabetes and pre‐diabetes, organ transplant, chronic liver disease and chronic renal failure excluded.	Hospital preventative health clinic (health care service)	20–39 yrs 53.77 (19.02) 40–59 yrs 55.64 (17.94) > 60 yrs 57.92 (18.51)	1 [≤ 11.36]	9.8 [11.92–34.07]	28.1 [34.64–49.97]	56 [50–85]	5.1 [> 85]	[91]
Japan	Ihara et al.	2004	Cross‐sectional study	176 female Japanese university students, 19–26 years. No history of smoking, regular medications, supplements or alcohol consumption.	University (community)	61.32 (13.63)	53.4 [≤ 39.75]			46.6 [> 39.75]		[71]
China	Ma et al.	2004	Cross‐sectional study	1163 healthy pregnant women from 4 sites throughout rural and city areas, non‐smoking, non‐drinking, nil iron supplementation. Aged 20–35 years, in third trimester of pregnancy. Community medical centres.	Population cohort pregnant (health care service)	Women Hb ≤ 100 16.53 (16.53) Hb 101‐119 20.46 (16.61) ≥ 120 28.5 (20.96)	36.89 [< 22.71]		63.11 [≥ 22.71]			[92]
China	Ma et al.	2009	Cross‐sectional study	734 healthy pregnant women in their third trimester, aged 20–35 years from 4 regions. Community medical centres.	Population cohort pregnant (health care service)	Women Hb < 110 g/L 17.09 (14.71) ≥ 110 22.03 (18.11)	Total 65.5 Hb < 110 69.2 Hb≥ 110 61 [< 22.71]		Total 34.5 [≥ 22.71]			[93]
China	Wang et al.	2018	Prospective cohort study	735 healthy pregnant women in their third trimester, aged 20–35 years from 4 regions.	Population ‐ rural (community)	Nil data	21 (ranging 5% in winter to 35% spring) [≤ 28]		79 [> 28]			[94]
Malaysia	Pirabbasi et al.	2013	Cross‐sectional study	97 male patients with COPD attending outpatient department, of any age or smoking status in final analysis. People with rheumatoid arthritis, tuberculosis and bronchitis were excluded.	Respiratory clinic (outpatient)	Nil data	Total 86.6 35‐50 yrs 83.3 ≥ 60 yrs 88.1. [< 22.7]		Total 13.4 35‐50 yrs 16.7 ≥ 60 yrs 11.9. [22.71‐85.17]			[95]
Philippines	Sia Su & Kayali	2008	Cross‐sectional study	49 motorized tricycle drivers in Sucat, Paranaque City and Alabang Hills in Manila. Mean age 37.8 (10.2) years.	City workers (community)	Nil data	Total 79.6 Sucat worker 62.5 Alabang Hills workers 96 [< 39.75]			Total 10.4 [39.97–113.56]		[19]
